# The association between controlling nutritional status and postoperative pulmonary complications in patients with colorectal cancer

**DOI:** 10.3389/fnut.2024.1425956

**Published:** 2025-01-13

**Authors:** Yafang Li, Chuang Nie, Na Li, Jieying Liang, Ning Su, Chunhua Yang

**Affiliations:** 1 Department of Intensive Care Unit, Biomedical Innovation Center, The Sixth Affiliated Hospital of Sun Yat-sen University, Guangzhou, China; 2 Department of Hematopathology, Biomedical Innovation Center, The Sixth Affiliated Hospital of Sun Yat-sen University, Guangzhou, China

**Keywords:** postoperative pulmonary complications, colorectal cancer, controlling nutritional status, survival, surgical outcome

## Abstract

**Background:**

Postoperative pulmonary complications (PPCs) significantly impact surgical outcomes, and Controlling Nutritional Status (CONUT) score, a simple and easily available nutritional score, has been demonstrated to be significantly associated with postoperative patient outcomes and complications, including PPCs. However, there are few studies that specifically focus on patients undergoing radical surgery for colorectal cancer (CRC).

**Methods:**

We retrospectively analyzed the clinical data of 2,553 patients who underwent radical surgery for CRC at the Sixth Affiliated Hospital of Sun Yat-sen University. Patients were divided into three groups: normal nutrition group (CONUT≤1), mild malnutrition group (2 ≤ CONUT≤4), and moderate-to-severe malnutrition group (CONUT≥5). Risk factors for PPCs and all-cause mortality were evaluated by multivariate regression. In addition, we assessed surgical outcomes including ICU admission, hospital stay, 1-year mortality and tumor-related mortality.

**Results:**

The incidence of PPCs was 9.0% (*n* = 230). Multiple regression showed that the higher the CONUT score, the higher the risk of PPCs (mild malnutrition group vs. normal nutrition group, OR: 1.61, 95% CI: 1.18–2.20, *p* = 0.003; moderate-to-severe malnutrition group vs. normal nutrition group, OR: 2.41, 95% CI: 1.51–3.84, *p* < 0.001). All-cause mortality was significantly higher in moderate-to-severe malnutrition group than that in normal nutrition group, HR: 1.88, (95% CI: 1.34–2.62, *p* < 0.001). Older age, male sex, chronic heart disease, open surgery, blood transfusion during surgery, distant metastasis of tumor and colon tumor were all risk factors for PPCs. Furthermore, the malnutrition groups had poor surgical outcomes including postoperative pneumonia (mild vs. normal nutrition, OR: 1.64, 95% CI: 1.07–2.52, *p* = 0.024; moderate-to-severe vs. normal nutrition, OR: 2.51, 95% CI: 1.36–4.62, *p* = 0.00), ICU admission (mild vs. normal nutrition, OR: 2.16, 95% CI: 1.31–3.56, *p* = 0.002; moderate-to-severe vs. normal nutrition, OR: 3.86, 95% CI: 2.07–7.20, *p* < 0.001), hospital stay ≥14 days (mild vs. normal nutrition, OR: 1.30, 95% CI: 1.08–1.56, *p* = 0.006) and 1-year mortality (mild vs. normal nutrition, HR: 1.65, 95% CI: 1.11–2.46, *p* = 0.014; moderate-to-severe vs. normal nutrition, HR: 2.27, 95% CI: 1.28–4.02, *p* = 0.005).

**Conclusion:**

The preoperative CONUT score is a potential indicator for predicting PPCs and surgical outcomes in CRC patients.

## 1 Introduction

Postoperative pulmonary complications (PPCs) are associated with increased mortality rates and prolonged hospital stays for patients who undergo surgeries, whether thoracic or nonthoracic (1–3), and the incidence of PPCs varies according to surgical sites (4). For abdominal surgery in particular, approximately 3.0 to 30.0% patients develop PPCs according to previous studies (5–9). There are already some prediction models, such as the ARISCAT model, used to predict the occurrence of PPCs in surgical patients (4), but these models require many different input factors to be known before surgery, making their practical value uncertain. Also, these models miss an important aspect of preoperative evaluation: malnutrition. Preoperative malnutrition, however, is becoming increasingly acknowledged as a key factor in the risk of PPCs (10, 11).

The Controlling Nutritional Status (CONUT) score, known for its simplicity and objectivity, has emerged as a valuable tool for assessing nutritional status in hospital patients (12). Some studies have shown that elevated CONUT scores are associated with higher risks of PPCs after surgery in gastric cancer (GC), bronchiectasis, and non-small cell lung cancer (NSCLC) patients (13–15). However, there is currently a lack of studies that specifically focus on the occurrence of PPCs in colorectal cancer (CRC) patients. Therefore, this study explores the relationship between the CONUT score and PPCs after CRC surgery. Other unfavorable postoperative outcomes such as hospital days, ICU admission, and mortality are also examined.

## 2 Materials and methods

### 2.1 Study design and patient population

This study is a single-center retrospective study, and the research protocol was approved by the Ethics Committee of the Sixth Affiliated Hospital of Sun Yat-sen University. Patients who were diagnosed with CRC and underwent surgery at the hospital between January 1, 2018, and December 31, 2019, were included.

**Figure 1:**
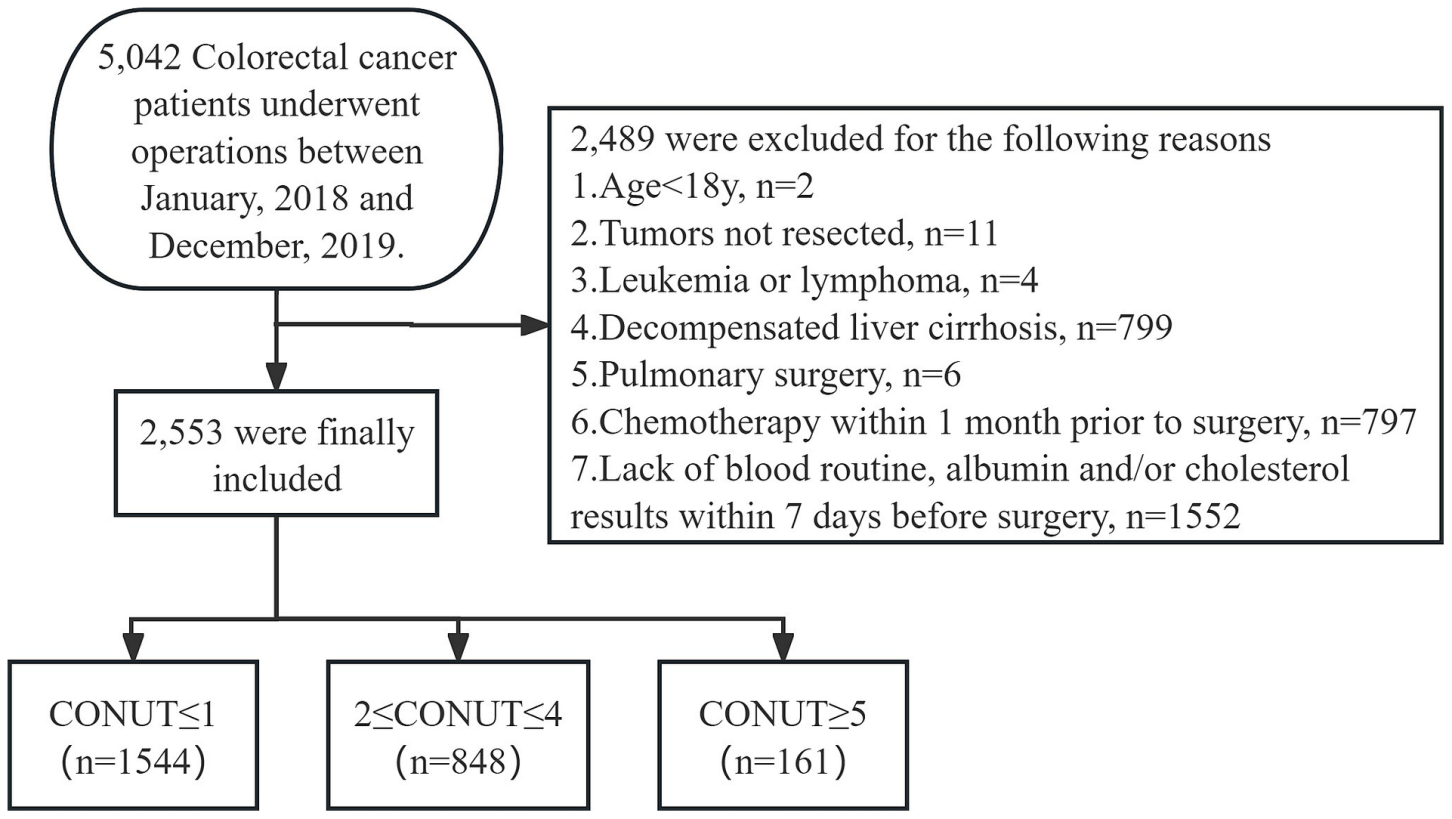
Flow diagram for 2,553 consecutive patients who met the inclusion criteria. PPCs: postoperative pulmonary complications; CONUT: controlling nutritional status.

The exclusion criteria were: (1) patients under 18 years of age, (2) patients with tumors not resected during surgery, (3) patients with leukemia or lymphoma, (4) patients with decompensated liver cirrhosis, (5) patients who underwent pulmonary surgery during operation, (6) patients who received chemotherapy within 1 month prior to surgery, and (7) patients lack of blood routine, albumin and/or cholesterol values within 7 days before surgery, as shown in Figure 1.

### 2.2 Clinical data

This study collected the data as follows. (1) Demographic data: age and gender. (2) Clinical data: body mass index (BMI), hypertension, diabetes, cardiovascular disease, chronic obstructive pulmonary disease (COPD), ischemic stroke, and history of previous abdominal surgery. (3) Surgery-related data: prophylactic antibiotics use, preoperative bowel preparations (use of enema/laxative), surgical approach (open/laparoscopic), intraoperative blood transfusion, enterostomy, tumor-node-metastasis (TNM) classification, and tumor location. (4) Laboratory data: white blood cells, lymphocytes, hemoglobin, albumin, cholesterol, and estimated glomerular filtration rate (eGFR). BMI was calculated as weight divided by height squared, and the eGFR was calculated using the CKD-EPI formula (16). Demographic and clinical data were obtained within 24 h of admission, and laboratory data were collected from valid results within the 7 days before surgery.

### 2.3 Definitions

Postoperative complications were recorded, the PPCs in our study were defined as one or more of the following complications within 1 month after surgery: pneumonia, respiratory failure, atelectasis, pleural effusion, and pneumothorax. The exact definitions are as follows: (1) Pneumonia: an infection of one or both lungs and diagnosed when the radiological criteria and clinical findings were met: (1) new or progressive and persistent infiltrate, consolidation or opacity, and cavitation, on chest X-ray or chest computed tomography (CT) and (2) fever (>38°C) with no other recognized cause and raised (≥12,000/mm^3^) or decreased (<4,000/mm^3^) white blood cell count. (2) Respiratory failure: arterial oxygen partial pressure (PaO_2_) less than 8 kPa (60 mmHg), a PaO_2_:FiO_2_ ratio less than 40 kPa (300 mmHg), or arterial oxygen saturation (SpO_2_) measured by pulse oximetry less than 90%. (3) Atelectasis: lung opacification observable on imaging, accompanied by a shift of the mediastinum, hilum, or hemidiaphragm toward the affected area. (4) Pleural effusion: blunting of the costophrenic angle on imaging, which may be accompanied by displacement of adjacent anatomical structures. (5) Pneumothorax: air within the pleural cavity, accompanied by absence of vascular markings surrounding the visceral pleura on imaging.

**Figure 2:**
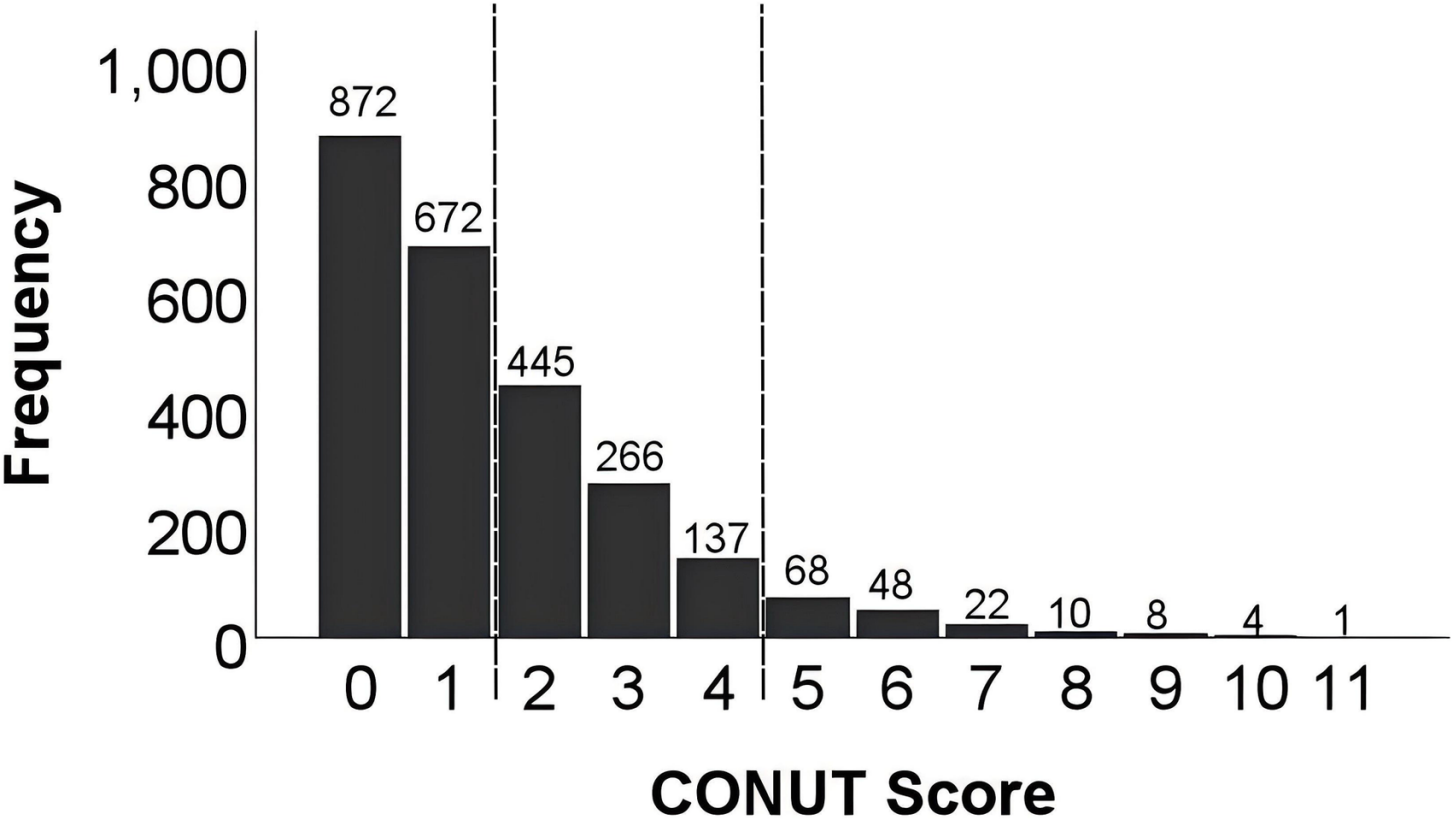
Frequency distribution of CONUT scores. Study population distribution according to CONUT score. 1,544 (60.5%) patients belonged to the CONUT ≤1 group, 848 (33.2%) patients belonged to the 2 ≤ CONUT ≤4 group, and 161 (6.3%) patients belonged to the CONUT ≥5 group. CONUT: controlling nutritional status.

The CONUT score, initially introduced by Ignacio de Ulibarri et al., serves as a tool for nutritional screening utilizing three laboratory parameters: serum albumin, lymphocyte count, and total cholesterol. This scoring system assigns points based on specific ranges for each parameter. For serum albumin, the ranges and corresponding points are: ≥ 3.50 g/dL (0 points), 3.00–3.49 g/dL (2 points), 2.50–2.99 g/dL (4 points), and < 2.50 g/dL (6 points). Lymphocyte count is scored as follows: ≥ 1,600/ml (0 points), 1,200-1,599/ml (1 point), 800–1,199/ml (2 points), and < 800/ml (3 points). Total cholesterol scoring is scored as: ≥ 180 mg/dL (0 points), 140–179 mg/dL (1 point), 100–139 mg/dL (2 points), and < 100 mg/dL (3 points). The total of these scores makes the CONUT score, with higher scores indicative of poorer nutritional status: normal (0–1); mild malnutrition (2–4); moderate malnutrition (5–8) and severe malnutrition (9–12) (12). According to the definition above, we divided our patients into 4 groups and found that the severe malnutrition group represents only 0.5% (*n* = 13) of the population (Figure 2). Hence, we combined the latter two groups and divided all the patients into three groups for analyses: normal nutrition group (CONUT≤1), mild malnutrition group (2 ≤ CONUT≤4), and moderate-to-severe malnutrition group (CONUT≥5) (Figure 2).

### 2.4 Follow-up

Follow-up visits were scheduled every 3 months within the first year after surgery, every 6 months for the subsequent 2–3 years, and annually thereafter for more than 3 years. Follow-up was conducted through outpatient visits, medical records review, letters, phone calls, and online communication.

### 2.5 Statistical analysis

Using SPSS Statistics Version 29 (IBM Co., Armonk, NY, United States), continuous data were expressed as mean ± standard deviation and analyzed using independent samples t-tests and ANOVA. Categorical data were presented as frequencies (percentages) and analyzed using Chi-squared tests and ANOVA. Multivariate logistic regression was conducted to determine independent predictors of the PPCs, and multivariate Cox regression was used to determine independent predictors of all-cause mortality. All variables with *p* < 0.1 in univariate analysis were then included in the multivariate analysis. Survival curves were calculated using the Kaplan–Meier method, and variations between curves were compared using the log-rank test. We used two-tailed *p* values in all statistical tests; *p* values <0.05 were considered to indicate statistically significant test results.

## 3 Results

### 3.1 Patient characteristics

A total of 2,553 patients were included in this study and classified into three groups by CONUT score; 1,544 (60.5%) patients belonged to normal nutrition (CONUT≤1) group, 848 (33.2%) belonged to mild malnutrition (2 ≤ CONUT≤4) group, and 161 (6.3%) belonged to moderate-to-severe malnutrition (CONUT≥5) group (Figure 2). Patient’s demographic and perioperative data are shown in Table 1; the average patient age was 59.55 years and 1,550 (60.7%) of the patients were male. Mean follow-up duration was 39.82 months. 363 (14.2%) patients underwent open surgery (compared to laparoscopic surgery), and 577 (22.6%) required an enterostomy. For tumor location, 1,114 (43.6%) were located in the rectum, 1,370 (53.7%) were located in the colon, and 69 (2.7%) were multifocal (Table 1).

**Table 1:** Patient characteristics.

Characteristics	Total (*n* = 2,553)	CONUT score			*p*-value
		≤ 1 (*n* = 1,544)	2 ~ 4 (*n* = 848)	≥ 5 (*n* = 161)	
Age, year	59.55 ± 12.55	58.28 ± 11.66	60.31 ± 13.40	67.81 ± 12.66	<0.001
Male, *n* (%)	1,550 (60.71)	915 (59.26)	532 (62.74)	103 (63.98)	0.171
BMI < 18.5, *n* (%)	237 (9.28)	105 (6.80)	99 (11.67)	33 (20.50)	<0.001
Comorbidities					
Heart disease, *n* (%)	108 (4.23)	49 (3.17)	42 (4.95)	17 (10.56)	<0.001
Diabetes mellitus, *n* (%)	250 (9.79)	135 (8.74)	98 (11.56)	17 (10.56)	0.081
Hypertension, *n* (%)	463 (18.14)	252 (16.32)	172 (20.28)	39 (24.22)	0.006
Cerebral ischemic stroke, *n* (%)	42 (1.65)	17 (1.10)	16 (1.89)	9 (5.59)	<0.001
COPD, *n* (%)	208 (8.15)	124 (8.03)	65 (7.67)	19 (11.80)	0.206
History of abdominal surgery, *n* (%)	278 (10.89)	149 (9.65)	104 (12.26)	25 (15.53)	0.022
Preoperative preparations					
Prophylactic antibiotics, *n* (%)	1,437 (56.29)	851 (55.12)	492 (58.02)	94 (58.39)	0.336
Preoperative bowel preparation, *n* (%)	2,479 (97.10)	1,511 (97.86)	823 (97.05)	145 (90.06)	<0.001
Intra-operative factors					
Open surgery, *n* (%)	363 (14.22)	172 (11.14)	134 (15.80)	57 (35.40)	<0.001
Enterostomy, *n* (%)	577 (22.60)	293 (18.98)	228 (26.89)	56 (34.78)	<0.001
Blood transfusion, *n* (%)	106 (4.15)	22 (1.42)	61 (7.19)	23 (14.29)	<0.001
Laboratory data					
White blood cell count, 10^9^/L	6.40 ± 2.09	6.56 ± 1.73	6.02 ± 2.38	6.80 ± 3.06	<0.001
Lymphocyte, 10^9^/L	1.71 ± 0.61	1.99 ± 0.53	1.33 ± 0.46	1.08 ± 0.43	<0.001
Hemoglobin, g/L	123.03 ± 22.91	129.79 ± 18.82	115.90 ± 24.04	95.68 ± 20.83	<0.001
Total cholesterol, mg/dL	191.77 ± 41.83	207.97 ± 35.58	171.88 ± 37.29	141.14 ± 33.74	<0.001
Albumin, g/dL	4.02 ± 0.48	4.20 ± 0.36	3.88 ± 0.44	3.14 ± 0.37	<0.001
eGFR, ml/(min·1.73m^2^)	88.27 ± 17.27	88.27 ± 16.13	88.87 ± 18.30	85.14 ± 21.58	0.043
Tumor invasion					
Tis, T1, T2	681 (26.67)	464 (30.05)	202 (23.82)	15 (9.32)	<0.001
T3, T4	1872 (73.33)	1,080 (69.95)	646 (76.18)	146 (90.68)	
Lymph node invasion					
N0	1,537 (60.20)	937 (60.69)	497 (58.61)	103 (63.98)	0.367
N1, N2	1,016 (39.80)	607 (39.31)	351 (41.39)	58 (36.02)	
Distant metastasis					
M0	2,347 (91.93)	1,459 (94.49)	744 (87.74)	144 (89.44)	<0.001
M1	206 (8.07)	85 (5.51)	104 (12.26)	17 (10.56)	
Location of tumor, *n* (%)					
Rectum	1,114 (43.63)	717 (46.44)	343 (40.45)	54 (33.54)	0.004
Colon	1,370 (53.66)	785 (50.84)	482 (56.84)	103 (63.98)	
Multifocal	69 (2.70)	42 (2.72)	23 (2.71)	4 (2.48)	
End Point, *n* (%)					
ICU admission, *n* (%)	102 (4.00)	28 (1.81)	47 (5.54)	27 (16.77)	<0.001
Hospital stay ≥14 days, *n* (%)	1,690 (66.20)	968 (62.69)	602 (70.99)	120 (74.53)	<0.001
1-year mortality, *n* (%)	119 (4.66)	46 (2.98)	54 (6.37)	19 (11.80)	<0.001
Tumor-related mortality, *n* (%)	283 (11.08)	144 (9.33)	106 (12.50)	33 (20.50)	<0.001
Overall mortality, *n* (%)	360 (14.10)	168 (10.88)	141 (16.63)	51 (31.68)	<0.001
PPCs, *n* (%)	230 (9.01)	92 (5.96)	101 (11.91)	37 (22.98)	<0.001

CONUT, controlling nutritional status; BMI, body mass index; COPD, chronic obstructive pulmonary disease; eGFR, estimated glomerular filtration rate; ICU, intensive care unit; PPCs, postoperative pulmonary complications.

Compared to the normal nutrition group, the malnutrition groups had older mean age, higher rate of BMI < 18.5, higher rates of cardiovascular disease, hypertension, and cerebral ischemic stroke, and was more likely to have a history of abdominal surgery (*p* < 0.05). Open surgery, enterostomy, and blood transfusion were more frequently performed in both malnutrition groups than in the normal nutrition group as well (*p* < 0.05). Furthermore, lymphocytes count, hemoglobin, albumin, and cholesterol were all significantly lower in the malnutrition groups (*p* < 0.05). The malnutrition groups both had a higher proportion of patients with tumor invasion T3/T4, M1 stage cancer (*p* < 0.001; Table 1).

With the increase of CONUT score, the incidence of the adverse postoperative outcomes gradually increased in normal nutrition, mild malnutrition, and moderate-to-severe malnutrition group; PPCs (5.96% vs. 11.91% vs. 22.98%, *p* < 0.001), ICU admission (1.81% vs. 5.54% vs. 16.77%, *p* < 0.001), Hospital stay ≥14 days (62.69% vs. 70.99% vs. 74.53%, *p* < 0.001), 1-year mortality (2.98% vs. 6.37% vs. 11.80%, *p* < 0.001), Tumor-related mortality (9.33% vs. 12.50% vs. 20.50%, *p* < 0.001), and Overall mortality (10.88% vs. 16.63% vs. 31.68%, *p* < 0.001; Table 1).

### 3.2 The occurrence of PPCs in different CONUT groups

Among the 2,553 patients, a total of 230 (9.0%) patients developed 401 cases of PPCs. Pleural effusion (6.2%) was the most common PPC, followed by pneumonia (4.4%) and atelectasis (3.3%). The incidence of PPCs in normal nutrition, mild malnutrition, and moderate-to-severe malnutrition group were 6.0, 11.9, 23.0%, respectively (*p* < 0.001). With the increase of CONUT score, the incidence of complications gradually increased in normal nutrition, mild malnutrition, and moderate-to-severe malnutrition group; pneumonia (2.8% vs. 5.7% vs. 11.8%, *p* < 0.001), respiratory failure (1.0% vs. 2.1% vs. 7.5%, *p* < 0.001), atelectasis (2.1% vs. 4.5% vs. 7.5%, *p* < 0.001), and pleural effusion (4.5% vs. 8.0% vs. 13.7%, *p* < 0.001; Table 2).

**Table 2:** Postoperative pulmonary complications between the high and low CONUT groups.

Postoperative pulmonary complications	Total (*n* = 2,553)	CONUT score			*p*- value
		≤ 1 (*n* = 1,544)	2 ~ 4 (*n* = 848)	≥ 5 (*n* = 161)	
Total	230 (9.0)	92 (6.0)	101 (11.9)	37 (23.0)	<0.001
Pneumonia	111 (4.4)	44 (2.8)	48 (5.7)	19 (11.8)	<0.001
Respiratory failure	46 (1.8)	16 (1.0)	18 (2.1)	12 (7.5)	<0.001
Atelectasis	83 (3.3)	33 (2.1)	38 (4.5)	12 (7.5)	<0.001
Pleural effusion	159 (6.2)	69 (4.5)	68 (8.0)	22 (13.7)	<0.001
Pneumothorax	2 (0.1)	0	2(0.1)	0	–

CONUT, controlling nutritional status.

### 3.3 The relationship between CONUT score and PPCs

Multivariate logistic analysis shows, as CONUT scores increased, the risk of PPCs significantly increased gradually. Compared to normal nutrition group, mild malnutrition group had 1.61 times risk of PPCs (OR: 1.61, 95% CI: 1.18–2.20, *p* = 0.003), and moderate-to-severe malnutrition group was 2.41 times higher (OR: 2.41, 95% CI: 1.51–3.84, *p* < 0.001; Table 3). We also analyzed the relationship between CONUT score and PPCs when dividing the patients into four groups by CONUT score (0–1, 2–4, 5–8, and ≥ 9), the result was consistent with the above (Supplementary Table S1).

**Table 3:** Multivariate logistic analysis of risk factors for PPCs.

Variable	Univariate analysis		Multivariate analysis	
	Unadjusted OR (95% CI)	*p*- value	Adjusted OR (95% CI)	*p*- value
CONUT score				
≤ 1	1.00		1.00	
2 ~ 4	2.13 (1.59–2.87)	<0.001	1.61 (1.18–2.20)	0.003
≥ 5	4.71 (3.08–7.19)	<0.001	2.41 (1.51–3.84)	<0.001
Characteristics				
Age	1.04 (1.02–1.05)	<0.001	1.03 (1.02–1.04)	<0.001
Male	1.44 (1.07–1.92)	0.014	1.62 (1.19–2.20)	0.002
BMI < 18.5	1.04 (0.65–1.64)	0.877		
Comorbidities				
Heart disease	2.42 (1.46–4.01)	0.001	1.84 (1.07–3.18)	0.028
Diabetes mellitus	1.69 (1.15–2.50)	0.008		
Hypertension	1.48 (1.07–2.03)	0.018		
Cerebral ischemic stroke	3.25 (1.58–6.71)	0.001		
COPD	2.06 (1.38–3.07)	<0.001		
History of abdominal surgery	1.42 (0.96–2.10)	0.079		
Preoperative preparations				
Prophylactic antibiotics	1.23 (0.93–1.62)	0.142		
Preoperative bowel preparation	0.41 (0.23–0.74)	0.003		
Intra-operative factors				
Open surgery	2.63 (1.79–3.57)	<0.001	1.91 (1.35–2.69)	<0.001
Enterostomy	1.43 (1.05–1.93)	0.021		
Blood transfusion	3.78 (2.39–5.98)	<0.001	2.46 (1.49–4.08)	<0.001
Tumor invasion				
Tis, T1, T2	1.00			
T3, T4	1.81 (1.27–2.58)	0.001		
Lymph node invasion				
N0	1.00			
N1, N2	1.25 (0.95–1.65)	0.106		
Distant metastasis				
M0	1.00		1.00	
M1	2.64 (1.81–3.85)	<0.001	2.13 (1.41–3.20)	<0.001
Location of tumor				
Rectum	1.00			
Colon	1.69 (1.26–2.26)	<0.001	1.55 (1.14–2.10)	0.005
Multifocal	2.11 (1.01–4.42)	0.048		

BMI: body mass index; COPD: chronic obstructive pulmonary disease; CONUT: controlling nutritional status; OR: odds ratio; CI: confidence interval.

Additionally, old age (OR: 1.03, 95% CI: 1.02–1.04, *p* < 0.001), male sex (OR: 1.62, 95% CI: 1.19–2.20, *p* = 0.002), cardiovascular disease (OR: 1.84, 95% CI: 1.07–3.18, *p* = 0.028), open surgery (OR: 1.91, 95% CI: 1.35–2.69, *p* < 0.001), intraoperative blood transfusion (OR: 2.46, 95% CI: 1.49–4.08, *p* < 0.001), the presence of distant tumor metastasis (OR: 2.13, 95% CI: 1.41–3.20, p < 0.001), and colon cancer (vs. rectal cancer, OR: 1.55, 95% CI: 1.14–2.10, *p* = 0.005) were significantly associated with PPCs (Table 3).

### 3.4 The relationship between CONUT score and overall mortality

In the multivariate Cox regression analysis, moderate-to-severe malnutrition group had a 1.88 times risk of mortality compared to normal nutrition group (HR: 1.88, 95% CI: 1.34–2.62, *p* < 0.001). However, there was no statistically significant difference between the mild malnutrition group and the normal nutrition group. Additionally, old age (HR: 1.03, 95% CI: 1.02–1.04, *p* < 0.001), previous history of abdominal surgery (HR: 1.35, 95% CI: 1.02–1.79, *p* = 0.037), open surgery (HR: 1.55, 95% CI: 1.21–2.00, *p* < 0.001), intraoperative enterostomy (HR: 1.86, 95% CI: 1.50–2.32, *p* < 0.001), tumor invasion T3/4 (HR: 1.73, 95% CI: 1.22–2.46, *p* = 0.002), lymph node invasion (HR: 2.80, 95% CI: 2.20–3.55, *p* < 0.001), and distant metastasis (HR: 4.28, 95% CI: 3.33–5.48, *p* < 0.001) were each significantly associated with overall mortality (Table 4).

**Table 4:** Cox regression analysis of risk factors for overall mortality.

Variable	Univariate analysis		Multivariate analysis	
	Unadjusted HR (95% CI)	*p*- value	Adjusted HR (95% CI)	*p*- value
CONUT score				
≤ 1	1.00		1.00	
2 ~ 4	1.61 (1.29–2.01)	<0.001	1.18(0.94–1.49)	0.154
≥ 5	3.42 (2.50–4.68)	<0.001	1.88(1.34–2.62)	<0.001
Characteristics				
Age	1.04 (1.02–1.06)	<0.001	1.03 (1.02–1.04)	<0.001
Male	1.04 (0.94–1.16)	0.435		
BMI < 18.5	1.55 (1.14–2.11)	0.005		
Comorbidities				
Heart disease	1.78 (1.18–2.69)	0.006		
Diabetes mellitus	1.28 (0.94–1.76)	0.123		
Hypertension	1.32 (1.03–1.69)	0.026		
Cerebral ischemic stroke	2.31 (1.30–4.10)	0.004		
COPD	1.24 (0.87–1.77)	0.224		
History of abdominal surgery	1.70 (1.29–2.25)	<0.001	1.35(1.02–1.79)	0.037
Preoperative preparations				
Prophylactic antibiotics	1.34 (1.08–1.65)	0.008		
Preoperative bowel preparation	0.44 (0.28–0.70)	<0.001		
Intra-operative factors				
Open surgery	2.41 (1.91–3.06)	<0.001	1.55(1.21–2.00)	<0.001
Enterostomy	2.41 (1.95–2.98)	<0.001	1.86(1.50–2.32)	<0.001
Blood transfusion	1.64 (1.08–2.51)	0.021		
Tumor invasion				
Tis, T1, T2	1.00		1.00	
T3, T4	3.22 (2.31–4.48)	<0.001	1.73(1.22–2.46)	0.002
Lymph node invasion				
N0	1.00		1.00	
N1, N2	3.74 (2.99–4.67)	<0.001	2.80(2.20–3.55)	<0.001
Distant metastasis				
M0	1.00		1.00	
M1	7.29 (5.79–9.18)	<0.001	4.28(3.33–5.48)	<0.001
Location of tumor				
Rectum	1.00			
Colon	1.00 (0.81–1.23)	0.988		
Multifocal	1.47 (0.85–2.55)	0.165		

BMI, body mass index; COPD, chronic obstructive pulmonary disease; CONUT, controlling nutritional status; HR, hazard ratio; CI, confidence interval.

### 3.5 The relationship between CONUT score and worse clinical outcomes

Multivariate logistic analysis revealed that the higher the CONUT score, the higher the risk of pneumonia (mild malnutrition group vs. normal nutrition group, OR: 1.64, 95% CI: 1.07–2.52, *p* = 0.024; moderate-to-severe malnutrition group vs. normal nutrition group, OR: 2.51, 95% CI: 1.36–4.62, *p* = 0.003); and the higher the risk of transfer to the ICU (mild vs. normal, OR: 2.16, 95% CI: 1.31–3.56, *p* = 0.002; moderate-to-severe vs. normal, OR: 3.86, 95% CI: 2.07–7.20, *p* < 0.001). Additionally, mild malnutrition group had higher risk of hospitalization exceeding 14 days (OR: 1.30, 95% CI: 1.08–1.56, *p* = 0.006) than normal nutrition group, while the moderate-to-severe malnutrition group does not show that difference (*p* = 0.129). Multivariate Cox regression analysis demonstrated that the higher the CONUT score, the higher the risk of 1-year mortality (mild malnutrition group vs. normal nutrition group, HR: 1.65, 95% CI: 1.11–2.46, *p* = 0.014; moderate-to-severe malnutrition group vs. normal nutrition group, HR: 2.27, 95% CI: 1.28–4.02, *p* = 0.005; Table 5).

**Table 5:** PPC incidence and surgical outcomes adjusted by CONUT.

	Univariate analysis		Multivariate analysis*	
	OR/HR (95% CI)	*p*- value	OR/HR (95% CI)	*p*- value
Pneumonia				
CONUT ≤1	1.00		1.00	
2 ≤ CONUT ≤4	2.05^a^ (1.35–3.11)	<0.001	1.64^a^ (1.07–2.52)	0.024
CONUT ≥5	4.56^a^ (2.59–8.02)	<0.001	2.51^a^ (1.36–4.62)	0.003
ICU admission				
CONUT ≤1	1.00		1.00	
2 ≤ CONUT ≤4	3.18^a^ (1.97–5.11)	< 0.001	2.16^a^ (1.31–3.56)	0.002
CONUT ≥5	10.91^a^ (6.25–19.05)	< 0.001	3.86^a^ (2.07–7.20)	<0.001
Hospital stay ≥ 14 days				
CONUT ≤1	1.00		1.00	
2 ≤ CONUT ≤4	1.46^a^ (1.22–1.74)	< 0.001	1.30^a^ (1.08–1.56)	0.006
CONUT ≥5	1.74^a^ (1.20–2.52)	0.003	1.34^a^ (0.92–1.97)	0.129
1-year mortality				
CONUT ≤1	1.00		1.00	
2 ≤ CONUT ≤4	2.18^b^ (1.47–3.23)	<0.001	1.65^b^ (1.11–2.46)	0.014
CONUT ≥5	4.19^b^ (2.45–7.15)	<0.001	2.27^b^ (1.28–4.02)	0.005
Tumor-related mortality				
CONUT ≤1	1.00		1.00	
2 ≤ CONUT ≤4	1.41^b^ (1.10–1.82)	0.007	0.99^b^ (0.77–1.29)	0.966
CONUT ≥5	2.58^b^ (1.77–3.77)	<0.001	1.38^b^ (0.90–2.11)	0.142

*Adjusted for age, gender, BMI, heart disease, diabetes mellitus, hypertension, cerebral ischemic stroke, COPD, history of abdominal surgery, prophylactic antibiotics, preoperative bowel preparation, laparoscopy, enterostomy, blood transfusion, tumor invasion, lymph node invasion, distant metastasis and location of tumor. CONUT, controlling nutritional status; ICU, intensive care unit; OR, odds ratio; HR, hazard ratio; CI, confidence interval. ^a^, OR; ^b^, HR.

**Figure 3:**
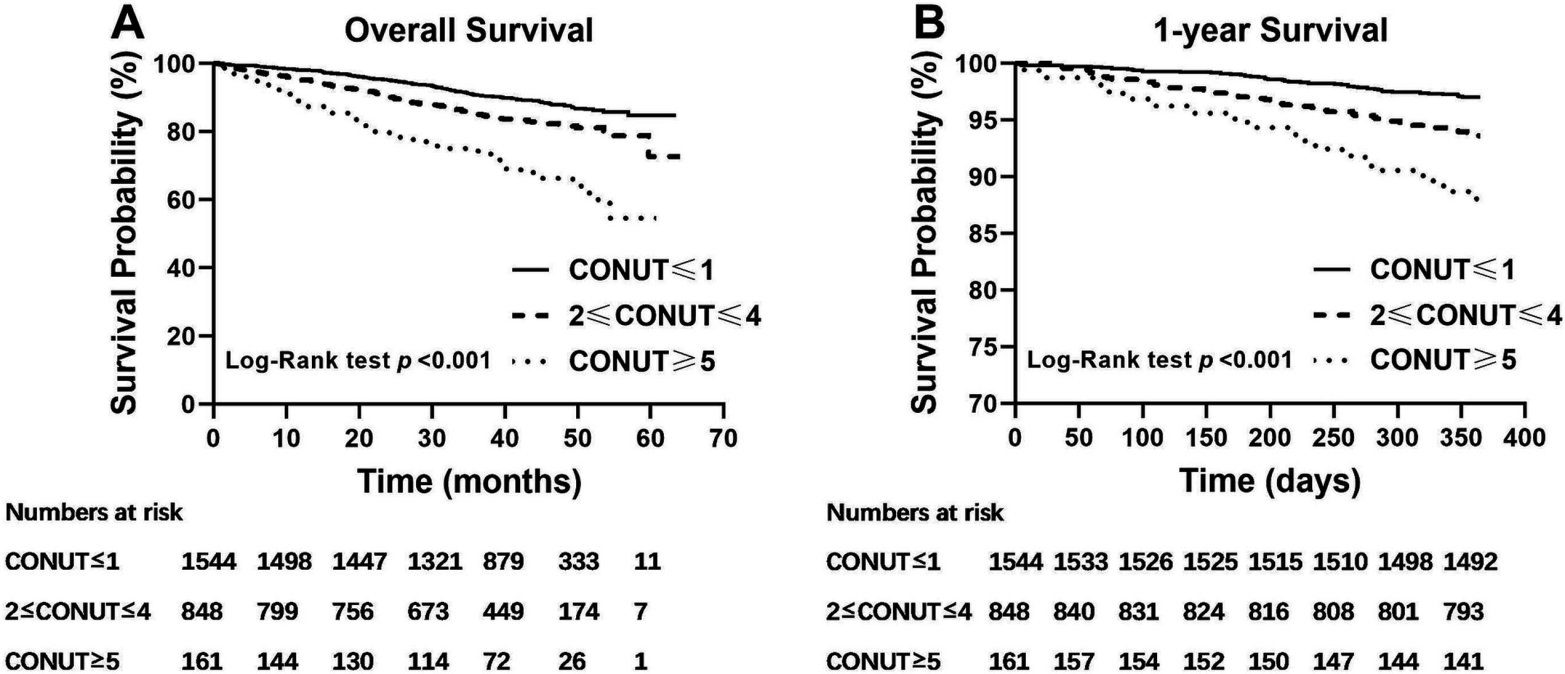
Kaplan–Meier survival curve for **(A)** overall and **(B)** 1-year survival according to CONUT status (log-rank test; *p* < 0.001). CONUT: controlling nutritional status.

Kaplan–Meier analysis was conducted and showed that the 1-year and overall survival rate among the normal nutrition, mild malnutrition and moderate-to-severe malnutrition groups significantly decreased gradually as the severity of malnutrition increased (*p* < 0.001; Figure 3).

## 4 Discussion

This study revealed that in CRC patients, as preoperative CONUT score increases, the risk of PPCs increases. Furthermore, the increase of CONUT score correlates with other adverse clinical outcomes, including overall mortality, 1-year mortality, ICU admission, and hospital stay ≥14 days.

As a tool for assessing nutritional status, the CONUT score has previously been reported to be a useful prognostic predictor for various postoperative complications. For example, Dong et al. showed that preoperative CONUT score could help to identify patients with a high possibility of malnutrition and postoperative complications (17). Qian et al. established the efficacy of the preoperative CONUT score as a practical tool for nutritional assessment, finding it to be predictive of short-term outcomes in gastric cancer (GC) patients following laparoscopy-assisted gastrectomy (18). Additionally, Rocans et al. highlighted the significant predictive utility of the CONUT score for flap complications after microvascular flap surgery (19). However, there are only a limited number of publications that specifically investigate the relationship between the CONUT score and PPCs. Lee et al. reported that preoperative CONUT score is an independent predictor of PPCs and 1-year mortality in patients with resectable NSCLC (14). In our study, postoperative pneumonia was one of the most important complications in PPCs, with an incidence of 4.4%. Postoperative pneumonia can lead to many adverse outcomes (9, 20). Our study suggests that postoperative CONUT as an indicator of malnutrition can also predict the occurrence of postoperative pneumonia. A study conducted by Sagawa et al. showed that preoperative malnutrition is an independent predictor of postoperative infections including pneumonia in patients with CRC (21). The result is similar to us.

The present study represents the first investigation to our knowledge into the relationship between the CONUT score and PPCs in patients undergoing CRC surgery. The potential mechanism underlying the relationship between the CONUT score and PPCs may be attributed to the score’s components, specifically levels of lymphocytes, cholesterol, and albumin. Low lymphocyte count could reflect a compromised immune response (22–24), which increases the risk of infections such as pneumonia. Research across various surgical populations has indicated that patients with preoperative lymphocytopenia experience a notably higher incidence of complications (25, 26). Similarly, altered cholesterol and albumin levels might indicate nutritional deficiencies or systemic inflammation (27–32). Hypoalbuminemia has been reported to have an effect on tissue healing or immune response impairment (33), thereby contributing to the development of PPCs. Moreover, evidence indicates that reduced cholesterol levels have a detrimental effect on postoperative outcomes by affecting antioxidant reserves and inflammatory response (34). Takagi et al. showed that a lower cholesterol level is associated with postoperative complications in patients undergoing gastrointestinal and hepatopancreatobiliary surgery as well (35). Cholesterol level also plays an important part in the prognosis of cancer patients because cell membrane fluidity is influenced by hypocholesterolemia, which relates to the mobility of cell surface receptors and the ability to transmit transmembrane signals (36).

According to the CONUT definition and nutrition status, patients in our study were divided into three groups: normal nutrition group (CONUT≤1), mild malnutrition group (2 ≤ CONUT≤4), and moderate-to-severe malnutrition group (CONUT≥5). With the increase of CONUT score, the severity of malnutrition increased, and the risk of PPCs, ICU transfer, hospitalization time ≥ 14 days and 1-year mortality increased gradually. As for overall mortality, there was no significant difference between mild malnutrition group and normal nutrition group. However, the risk in moderate-to-severe malnutrition group (CONUT≥5) was nearly twice as high as that in normal nutrition group. In previously studies conducted by Okamoto et al. and Liu et al., they divided the population into two groups based on cutoff points of CONUT≥4 and ≥ 5, respectively. They concluded that, the higher preoperative CONUT score relates to higher postoperative complications and higher overall mortality in CRC Patients (37, 38). This is consistent with the results of our study. In addition, a study involving 830 elderly patients with CRC divided patients into three groups by CONUT score (≤1, 2–3, ≥4). The study found that as CONUT score increased, the risk of overall mortality increased gradually (39). In another study, CRC patients were divided into four groups according to the CONUT definition (≤1, 2–4, 5–8, ≥9). The K-M analysis of overall survival and CONUT was performed. It was found that the overall survival rate tended to be lower in patients with higher COUNT score (40). All these results were consistent with us. That is, with the increase of CONUT score, the short-term complications rates and long-term overall mortality of patients increased.

Other factors, such as intraoperative blood transfusion may indicate intraoperative blood loss, which is already a known risk factor for PPCs in patients undergoing thoracoscopic lobectomy for NSCLC (41). Compared to minimally invasive techniques, open surgery typically involves larger incisions, longer recovery times, and reduced mobility, which all lead to greater risk for PPCs (42, 43). Furthermore, advanced age and later TNM stage have both been reported to be independent risk factors for PPCs in different study populations (42, 44).

This study has several limitations, however. First, due to its observational design, nutritional management, surgical, and anesthesia decisions were made by the clinicians, which may have led to potential variability across different cases. Second, this study excluded 1,552 patients due to missing data, and the rate of open surgery among the excluded patients was higher than that of the final patient cohort (18.4% vs. 14.2%, *p* < 0.001; Supplementary Table S2). Since our study confirms that open surgery was an independent risk factor for postoperative pulmonary complications (PPCs) (Table 3), this may result in a slightly lower incidence of PPCs reported in this study than what actually occurred. Third, as this study is a single-center retrospective study, further large-scale prospective studies are needed before the findings can be applied to guide clinical practice more generally.

## 5 Conclusion

In conclusion, we find that preoperative CONUT score is associated with PPCs and surgical outcomes in patients undergoing CRC surgery. Preoperative CONUT may therefore be a valuable prognostic factor for PPCs and surgical outcomes in CRC.

## Data Availability

The data analyzed in this study is subject to the following licenses/restrictions: The data are not publicly available due to their containing information that could compromise the privacy of research participants. Requests to access these datasets should be directed to Yafang Li, liyafang3@mail.sysu.edu.cn.
