# Supplementary material for: The association between controlling nutritional status and postoperative pulmonary complications in patients with colorectal cancer
Source: Front Nutr. 2025 Jan 13;11:1425956. doi: 10.3389/fnut.2024.1425956 (PMC11769804; doi:10.3389/fnut.2024.1425956)
Supplement: Supplementary file 1 [file Supplementary_file_1.docx]

Supplementary table 1. PPCs incidence adjusted by CONUT.

| Variable | Univariate analysis | | Multivariate analysis* | |
| --- | --- | --- | --- | --- |
|  | OR (95% CI) | P -value | OR (95% CI) | P -value |
| CONUT score |  |  |  |  |
| ≤ 1 | 1.00 |  | 1.00 |  |
| 2 ~ 4 | 2.134 (1.587 - 2.869) | <0.001 | 1.613 (1.183 - 2.201) | 0.003 |
| 5 ~ 8 | 4.354 (2.791 - 6.791） | <0.001 | 2.290 (1.409 - 3.724) | 0.001 |
| ≥ 12 | 9.864 (3.164 - 30.752） | <0.001 | 3.817 (1.152 - 12.646) | 0.028 |

*Adjusted for age, gender, body mass index, heart disease, diabetes mellitus, hypertension, cerebral ischemic stroke, chronic obstructive pulmonary disease, history of abdominal surgery, prophylactic antibiotics, preoperative bowel preparation, laparoscopy, enterostomy, blood transfusion, tumor invasion, lymph node invasion, distant metastasis and location of tumor. PPCs: postoperative pulmonary complications; CONUT: controlling nutritional status; OR: odds ratio; CI: confidence interval.

Supplementary table 2. Characteristics of patients excluded from the study.

|  | Final patients  (n=2553) | Excluded patients (n=1552) | Total  (n=4105) | P -value |
| --- | --- | --- | --- | --- |
| Age; year | 59.6 ± 12.5 | 60.2 ± 12.9 | 60.0 ± 12.7 | 0.104 |
| Sex; male | 1550 (60.7%) | 955 (61.5%) | 2505 (61.0%) | 0.621 |
| BMI＜18.5 | 237 (9.3%) | 150 (9.7%) | 387 (9.4%) | 0.849 |
| Heart disease | 108 (4.2%) | 85 (5.5%) | 193 (4.7%) | 0.068 |
| Diabetes mellitus | 250 (9.8%) | 168 (10.8%) | 418 (10.2%) | 0.312 |
| Hypertension | 463 (18.1%) | 335 (21.6%) | 798 (19.4%) | 0.007 |
| Cerebral ischemic stroke | 42 (1.7%) | 38 (2.4%) | 80 (1.9%) | 0.081 |
| COPD | 208 (8.2%) | 146 (9.4%) | 354 (8.6%) | 0.169 |
| History of abdominal surgery | 278 (10.9%) | 184 (11.9%) | 462 (11.3%) | 0.359 |
| Prophylactic antibiotics | 1437 (56.3%) | 778 (50.1%) | 2215 (54%) | 0.000 |
| Preoperative bowel preparation | 2479 (97.1%) | 1512 (97.4%) | 3991 (97.2%) | 0.559 |
| Open surgery | 363 (14.2%) | 286 (18.4%) | 649 (15.8%) | 0.000 |
| Blood transfusion | 106 (4.2%) | 73 (4.7%) | 179 (4.4%) | 0.431 |
| Tumor invasion |  |  |  |  |
| Tis, T1, T2 | 681 (26.7%) | 459 (29.6%) | 1140 (27.8%) | 0.048 |
| T3, T4 | 1872 (73.3%) | 1093 (70.4%) | 2965 (72.2%) |  |
| Lymph node invasion |  |  |  |  |
| N0 | 1537 (60.2%) | 962 (62.0 %) | 2499 (60.9%) | 0.169 |
| N1, N2 | 1016 (39.8%) | 590 (38.0 %) | 1606 (39.1%) |  |
| Distant metastasis |  |  |  |  |
| M0 | 2347 (91.9%) | 1431 (92.2%) | 3778 (92.0%) | 0.767 |
| M1 | 206 (8.1%) | 121 (7.8%) | 327 (8.0%) |  |
| PPCs | 230 (9.0%) | 145 (%) | 375 (9.1%) | 0.738 |
| Overall mortality | 360 (14.2%) | 245 (%) | 605 (14.7%) | 0.335 |

BMI: body mass index; COPD: chronic obstructive pulmonary disease; PPCs: postoperative pulmonary complications
